# Physician-Controlled Wire-Guided Cannulation of the Minor Papilla

**DOI:** 10.1155/2010/629308

**Published:** 2010-08-11

**Authors:** John T. Maple, Lilah Mansour, Tarek Ammar, Michael Ansstas, Gregory A. Coté, Riad R. Azar

**Affiliations:** ^1^Division of Digestive Diseases and Nutrition, University of Oklahoma Health Sciences Center, 920 Stanton L. Young Boulevard, WP 1345, Oklahoma City, Oklahoma 73117, USA; ^2^Division of Gastroenterology, Washington University School of Medicine, 660 South Euclid Avenue, Campus Box 8124, St. Louis, MO 63110, USA

## Abstract

*Background*. Minor papilla (MiP) cannulation is frequently performed using specialized small-caliber accessories. Outcomes data for MiP cannulation with standard-sized accessories are lacking. *Methods*. This is a case series describing MiP cannulation outcomes in consecutive patients treated by two endoscopists between July 2005 and November 2008 at two tertiary referral centers. MiP cannulation was attempted using a 4.4 Fr tip sphincterotome loaded with a 0.035^″^, 260 cm hydrophilic-tip guidewire, using a wire-guided technique under physician control. *Results*. 25 patients were identified (14 women, mean age 45). Procedure indications included recurrent acute pancreatitis in 16 patients (64%) and chronic pancreatitis in 2 (8%), among other indications. MiP cannulation was successful in 24 patients (96%). Sphincterotomy followed by pancreatic stent placement was performed in 21 patients (84%). Mild post-ERCP pancreatitis occurred in 3 patients (12%). *Conclusion*. Physician-controlled wire-guided MiP cannulation using a 4.4 Fr sphincterotome and 0.035^″^ guidewire is an effective and safe technique.

## 1. Introduction

Endoscopic access to the dorsal pancreatic duct may be sought in a variety of clinical situations, most commonly in patients with pancreas divisum associated with idiopathic recurrent acute pancreatitis (IRAP) or chronic pancreatitis. Minor papilla (MiP) cannulation may be challenging, and historically experts have often advocated the use of specialty accessories (e.g., needle-tip catheters, ultrataper tip catheters) and small-caliber (e.g., 0.018′′ or 0.021′′) wires when approaching the MiP [1–4]. However, pancreas divisum may be an unanticipated finding during endoscopic retrograde cholangiopancreatography (ERCP), and the benefit of changing to a specialty platform is uncertain. In our experience, the MiP may be routinely cannulated using a standard pull sphincterotome and 0.035′′ guidewire, if a wire-guided technique is employed. The objective of our study was to describe the efficacy and safety of MiP cannulation using standard accessories and a wire-guided technique.

## 2. Methods

### 2.1. Patients

All patients who underwent attempted MiP cannulation by 2 endoscopists (R. R. Azar, and J. T. Maple) at 2 tertiary care medical centers between July 2005 and November 2008 were identified using searchable endoscopy databases. Both endoscopists exclusively employed the cannulation technique described below. Demographic and procedural data were abstracted, and electronic medical records were reviewed for clinical followup, including complications. This study was approved by the institutional review boards for our medical centers.

### 2.2. Procedure Methods

Patients were sedated using either moderate sedation (midazolam and meperidine) directed by the endoscopist or deep sedation (propofol) administered by a nurse anesthetist. A duodenoscope (TJF-160F or TJF-160VF, Olympus America, Melville, NY) was used for all examinations. Glucagon and secretin were used at the endoscopist's discretion to assist with duodenal aperistalsis and identification of the MiP orifice. After MiP identification, generally a “long scope” position was utilized for cannulation, though occasionally a “very short” scope position was used if adequately stable. 

**Figure 1:**
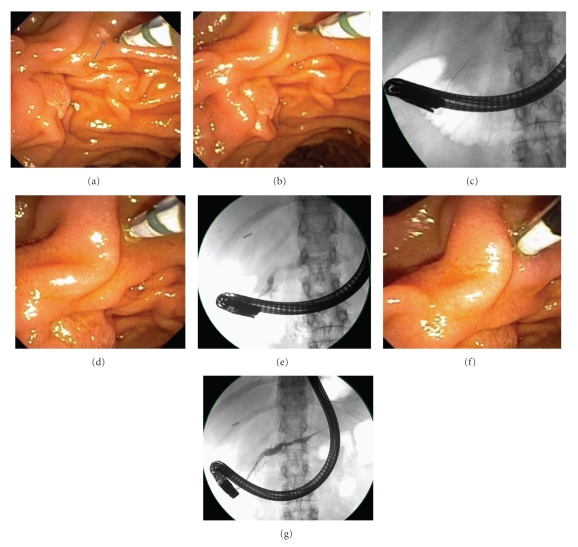
Cannulation technique: (a) a pull-type sphincterotome loaded with a 0.035′′ guidewire is positioned adjacent to the minor papilla (arrow). (b) The wire is used to cannulate the papillary os. (c) The wire is advanced 20 mm, achieving superficial wire cannulation. (d, e) The sphincterotome is lightly impacted on the minor papilla and a dorsal pancreatogram is then obtained. (f, g) Deep wire and device cannulation are attained and more robust dorsal ductography is performed.

Cannulation was attempted with a short-nose traction sphincterotome loaded with a 0.035′′, 260 cm guidewire in all cases. In the vast majority of cases, a 4.4 Fr tip Autotome 44 (Boston Scientific, Natick, MA) and 0.035′′ Hydra Jagwire (Boston Scientific, Natick, MA) were employed. With the sphincterotome hovering in the duodenum, the endoscopist advances the guidewire into the os of the MiP and subsequently gently advances the wire 10–20 mm or until any resistance is met, using fluoroscopic guidance (Figure 1). 

The sphincterotome is then advanced until it enters or abuts the MiP, and contrast is injected for dorsal ductography. In cases requiring therapeutic intervention, the wire is then advanced more deeply into the dorsal duct and resecured, and the sphincterotome is passed into the dorsal duct. When the orifice does not permit passage of the sphincterotome, a needle knife is used to perform an access sphincterotomy alongside the guidewire. Pancreatic stents are placed into the dorsal duct in all procedures in which minor papillotomy is performed. 

## 3. Results

Twenty-five patients underwent attempted MiP cannulation between July 2005 and November 2008. Patient demographics and procedure indications are summarized in Table 1. 

Eighteen of the procedures were performed at Washington University in St. Louis (R. R. Azar) and seven at Oklahoma University Medical Center (J. T. Maple). Seventeen patients had pancreatic imaging (e.g., CT, magnetic resonance cholangiopancreatography (MRCP), and endoscopic ultrasound) prior to the ERCP that was available for the authors to review; pancreas divisum was suspected in 10 of these patients. In the remaining 8 patients, no imaging was available for the authors to review—most of these patients had undergone CT or MRCP elsewhere, but the study images were unavailable. In these latter cases, no mention was made of the presence of (or suspicion for) pancreas divisum on the radiology report.

Procedural findings and outcomes are summarized in Table 2. 

MiP cannulation was successful in 24 of 25 patients (96%). Secretin was administered in 9 patients (36%). A minor papillotomy was performed in 21 patients (84%), using a pull-type sphincterotomy in 19 patients and using a needle knife alongside a cannulated guidewire in 2 patients. A 5 Fr (*n* = 15) or 7 Fr (*n* = 6) stent was placed into the dorsal pancreatic duct in all 21 patients requiring minor papillotomy. The median procedure time, recorded in 18 patients, was 31 minutes. 

Three patients (12%) developed mild post-ERCP pancreatitis. An additional 3 patients (12%) experienced abdominal pain without hyperamylasemia. Eleven patients underwent repeat ERCP, generally for stent removal or exchange. In the remaining 10 patients in whom a stent was placed, follow-up abdominal radiography (usually at ten days) confirmed spontaneous stent migration. In the lone patient in whom cannulation failed, a different endoscopist not involved with this study achieved successful minor papilla cannulation at a 2nd ERCP by performing dorsal ductography with a needle-tip catheter, followed by deep cannulation using a tapered-tip cannula and 0.021′′ wire.

## 4. Discussion

Minor papilla cannulation remains challenging, even for experienced endoscopists. However, relatively high rates of cannulation success (86%–93%) may be ultimately achieved at experienced centers [1, 5–7]. When pancreas divisum is suspected prior to the case, dedicated accessories are often selected for MiP cannulation, such as needle-tip or highly tapered catheters and small-caliber wires. However, pancreas divisum may be an uncertain or unanticipated finding during an ERCP. Indeed, pancreas divisum was known a priori in only 40% of patients in this series prior to ERCP. During cases in which a standard 0.035′′ platform is already in use, it is unclear whether changing to a small-caliber specialty platform will increase MiP cannulation success. However, the use of additional wires and devices will clearly add expense and can be time consuming.

In this series we have demonstrated that a 4.4 Fr pull sphincterotome and 0.035′′ wire may be used for MiP cannulation with a high success rate (96%), when a physician-controlled wire-guided cannulating technique is employed. This approach also appears to be safe; the 12% rate of post-ERCP pancreatitis is similar to pancreatitis incidence in prior series of MiP access and papillotomy (8%–20%) [3–8]. Though undoubtedly historically utilized, this report is the first to our knowledge to describe and evaluate this technique.

Wire-guided biliary cannulation has also been recently studied. Metaanalyses of randomized controlled trials have demonstrated both an increase in cannulation success and a reduction in post-ERCP pancreatitis when a wire-guided technique is used, as compared to conventional device and contrast methods [9, 10]. It is possible that the same potential benefits may be also seen at the MiP, perhaps as a result of minimizing papillary trauma and contrast volume. However, implicit in the success of any wire-guided cannulating technique at either papilla is skillful handling of the guidewire. Cautious wire advancement, with close attention to both visual and tactile feedback, is critical to both the success and safety of the technique, as indiscriminant wire probing can induce papillary trauma, false submucosal passages, or trauma to a small pancreatic branch duct. In this series, only the endoscopist handled the wire; however, a highly experienced assistant may also be suitable for wire management.

This series is subject to several limitations. First, these data are retrospective. While the endoscopic databases are complete, the search strategies employed may be imperfect, with risk for missed cases including failures, despite our diligence. The retrospective nature of the study may also limit the accuracy of assessing adverse events, though at each institution, all patients are routinely contacted by telephone 1–3 days post ERCP. The number of patients described is small, and one or two additional failed cannulations would alter the success rate. Lastly, the two endoscopists in this series are interventional endoscopists with prior ERCP training beyond a standard fellowship. While this may affect the generalizability of these findings, it should be noted that patients requiring MiP intervention are frequently referred to endoscopists of comparable skill at tertiary centers.

In conclusion, physician-controlled wire-guided MiP cannulation using a 4.4 Fr sphincterotome and 0.035′′ guidewire is an effective and safe technique that may obviate the need for specialty accessories. However, prospective evaluation of this technique in a greater number of cases is needed to more accurately assess outcomes. 

## Figures and Tables

**Table 1:** Patient demographics and procedure indications.

	*N* = 25
Age (mean, range)	45, 12–78
Gender	14 F, 11 M
Indication	
IRAP	16 (64%)
CP	2 (8%)
Pseudocyst	2 (8%)
IAP	2 (8%)
Other	3 (12%)
Divisum known prior to ERCP?	10 (40%)

IRAP: idiopathic recurrent acute pancreatitis, CP: chronic pancreatitis, IAP: idiopathic acute pancreatitis, Other: pancreatic duct leak, pancreas divisum and pain only, and choledocholithiasis.

**Table 2:** Procedural findings and outcomes.

	*N* = 25
Cannulation success	24 (96%)
Anatomy	
Pancreas divisum	22 (88%)
Other*	3 (12%)
Pathologic findings	
Chronic pancreatitis	9 (36%)
Stones, strictures	
Pseudocyst(s)	5 (20%)
PD leak	1 (4%)
Minor papillotomy	21 (84%)
Dorsal PD stent	21 (84%)
Post-ERCP pancreatitis	3 (12%)

*Other: normal (1), pseudodivisum due to obstructing stone(s) in the ventral duct (1), unfused pancreatic ducts, yet ventral dominant—the dorsal duct ended blindly after 2 cm (1), PD: pancreatic duct.
